# The Future of St. Bartholomew's Hospital

**Published:** 1903-11-21

**Authors:** 


### THE FUTURE OF ST. BARTHOLOMEW'S HOSPITAL.

### SUPPRESSION OF THE EVIDENCE OF THE MEDICAL STAFF.

THERE appears to be no abatement of the interest which is being shown by the general public in the welfare of St. Bartholomew's Hospital at this critical moment in its history, and it is hard to believe that the question relative to its future was finally settled at the recent meeting of the General Court of Governors. It appears that some of the Governors were called upon, on that occasion, to make their decision, and ratify the proposals put forward by the Mansion House Committee in ignorance of certain facts having an important bearing on the issue. It is said that the evidence given before the Lord Mayor's Committee by the Medical Council was not placed before the whole of the Governors in its entirety, and if this was the case, it is certain that a grave mistake was made, and one which might justly be held to call for a reconsideration of the decision arrived at In order to make the matter clearer, it may not be altogether out of place to indicate what are the responsibilities of individual Governors. It is the usual custom among our voluntary hospitals for the ordinary routine management of each institution to be placed in the hands of a committee formed of a limited number of the Governors who, from the influence they possess, the time they have at their disposal, or for some other reason are specially fitted to have the guidance of the hospital entrusted to them. On the other hand, matters which are of such importance that they cannot well be regarded as falling within the purview of routine management are submitted to a general meeting, at which each individual Governor may have a voice and a vote.

Now the present position in which St. Bartholomew's Hospital is situated is without doubt a crisis in the history of that ancient institution. The question of its re-construction is one of the largest problems that our voluntary hospital system has up to this time had to face. Since St. Bartholomew's Hospital forms no exception to the rule of management described above, the ultimate decision of the question as to whether the scheme sanctioned by the Mansion House Committee was an adequate one and worthy to be carried into effect, or whether it should be rejected or modified, rested with the General Court of Governors and not with a relatively small number of officials. It follows from this that unless the General Court of Governors is to be considered a mere farce, every individual Governor ought to have had submitted to him all details having a direct bearing on the matter at issue, so that he would have time to peruse it in private, and weigh the matter well before being called upon to give his decision. This rule of procedure, we are informed was not carried out previous to the General Meeting of Governors which was held on the 5th instant, and at which the report of the Mansion House Committee was approved of, and official sanction thus obtained for carrying out a scheme which those who are in the best position to judge are quick to condemn as unworthy of the City and of the nation.

At the meeting referred to we are told that each Governor was handed a copy of certain resolutions and was asked to vote upon them without having any proper opportunities provided him for forming an opinion of his own upon the merits or the demerits of the propositions placed before him. On the other hand the mover of the resolutions, Mr. Cripps, urged upon the meeting the desirability of adopting the resolutions unanimously, on the ground that otherwise the public would hesitate to subscribe the £350,000 needed. Under these circumstances it is small wonder that the scheme propounded by the Mansion House Committee was adopted. The really vital portion of the evidence, namely, a full report of the views of the Medical Council as they were laid before the Mansion House Committee, are stated to have been withheld or only partially revealed. More than this, before the Lord Mayor's Committee was appointed the Medical Council called the attention of the Governors to the fact that the area possessed by St. Bartholomew's Hospital was not sufficient for a hospital of nearly 700 beds, and expressed the opinion that it was necessary to increase the site. The reports of the Medical Council embodying this opinion and including suggestions upon other matters have not, so it is said, yet been submitted in their entirety to each Governor. In this way, it would seem, a dangerous precedent has been set, which, if followed by voluntary hospitals, may be most harmful to their responsible management.

In order to make our contention clear it may be well to describe briefly the system upon which St. Bartholomew's Hospital is managed. In the first place, the general administration is under the authority of the Treasurer and four almoners. Next there is the House Committee, which meets once a month. Then there is the General Court of Governors, which meets once every three months, and at which every Governor may be present and is entitled to vote. There is a regulation in force by which no member of the visiting medical staff may become a Governor. The Medical Council, however, may send delegates to the meetings of the Governors, where they may express opinions, give explanations, and offer suggestions, but not vote. All official communications to the Governors from the Medical Council would be handed by its secretary, who is a member of the medical staff, to the clerk of the Governors who would, presumably, place it before the treasurer and almoners. If then, as is stated, the medical council have sent reports relative to plans for the future construction of St. Bartholomew's to the Governors of that institution, some of which reports are stated to fill many pages, how far, we ask, have such reports been circulated among the Governors? Have they been submitted to each member of the House Committee, and, if so, have they percolated to any extent beyond the House Committee among the general body of Governors?

St. Bartholemew's Hospital is particularly fortunate in having among the members of its Medical Council some of the leading lights of the medical and surgical world, and their counsel and advice in regard to matters which appertain to their spheres, should be given their proper importance, and indeed should rightly be the fundamental guides to the settlement of the question at issue. It is clear, however, that the Governors have decided to act in direct opposition to their counsel and to appeal to the public to supply them with money for a scheme which those best fitted to judge still maintain to be insufficient and inadequate to provide the City of London with a modern hospital. What the public now wishes to learn before supplying the necessary money is, on what grounds the recommendations of the Medical Council have been set on one side. If sufficiently convincing reasons are not forthcoming, is it to be expected that the benevolent will be willing to overlook the expert opinions of the eminent members of the Medical

Council of St. Bartholomew's Hospital which have been thus far disregarded by the management of that institution.

---

DEPUTATION TO THE LORD MAYOR.

A DEPUTATION of Governors of St. Bartholomew's Hospital waited upon the Lord Mayor at the Mansion House on Tuesday last, with a request that at some convenient date he would convene a meeting of the citizens for the purpose of raising the requisite funds for the new buildings and improvements needed at the hospital. The deputation consisted of Sir Trevor Lawrence, the treasurer, the Rev. Sir Borradaile Savory, Alderman Sir William Treloar, Mr. Alderman Alliston, Mr. George Baker, Mr. John Carey Lovell, Mr. G. Acton Davis, Mr. P. L. Blyth, Mr. E. Mulready Stone, Mr. John Smithers, Mr. W. W. Bramston, Mr. Henry Perrin, Mr. Richard Stapley, Mr. Bernard F. Harris, Mr. James Figgins, Mr. John R. Cooper, Mr. F. M. Fry, Mr. H. Attlee, Mr. W. D. Cronin, and Mr. W. H. Cross, the clerk of the hospital.

Sir Trevor Lawrence said it would be within the recollection of the Lord Mayor that his predecessor, Sir Marcus Samuel, summoned a Mansion House committee to report as to whether it was desirable to retain St. Bartholomew's Hospital on its present site, and, if so, whether any better scheme of rebuilding than that suggested by the Governors could be devised. That committee was thoroughly impartial, nine members being selected by the Lord Mayor and six by himself as treasurer. The committee, after a long, careful, and exhaustive inquiry, decided that it was impossible in the public interest to remove the hospital from its site, and that the additions to and rearrangement and improvement of the existing buildings were absolutely necessary for continued efficiency in the treatment of patients and for bringing the hospital up to the standard of modern sanitary and scientific requirements. They also affirmed that the reputation and character of the hospital had been fully vindicated, and that its administration had been conducted in a wise and enlightened spirit, with due regard to economy and the best interests of the patients. It was 150 years since any outside appeal had been made on behalf of St. Bartholomew's Hospital. What had happened to St. Bartholomew's had arisen in all or many of the older hospitals. They required to renew, alter, change, or add to their existing departments or buildings to make them more adaptable to the ever-increasing modern requirements of medical science. It was necessary in the case of St. Bartholomew's to rebuild much that was old, and to add many desirable buildings for its great work. The whole question had been gone into, and it was found absolutely impracticable to buy any more land. The Governors had bought and paid for the land which they acquired from Christ's Hospital nearly a quarter of a million. The provision of that large sum would reduce the income of the hospital by £9,000 per annum. As they had previously had a surplus income of £2,000 a year, that would create a deficiency of £7,000 per annum. Having spent so much upon the land, it was impossible, out of their funds, to supply any of the money needed for the new buildings, and, in this, they asked the citizens of London to help them. The Queen had given £1,000 and the great advantage of her gracious support and encouragement. The Prince of Wales, as their President, was interested in many other hospitals and in King Edward's Hospital Fund, but he was according them his sympathy and assistance, and would not disassociate himself from their appeal. The sum wanted was £300,000. They did not expect or require it all at once. The donations could be spread over three or four years, and the work proceeded with gradually. The Mansion House had always been the great centre of charity for the country at large, and to it the Governers naturally turned in the present emergency. They did not despair of success, seeing the splendid response which recently attended a similar appeal for Guy's Hospital. Like Guy's, they had suffered from agricultural depression, but in course of time they would get an increased income when certain leases fell in. As he had said, they could not devote any more of their corporate funds for the great object they had in view, and an appeal to the generosity of the public was inevitable.

Mr Alderman Alliston spoke strongly in support of the application.

The Lord Mayor, in reply, said he had received the deputation with great pleasure. He was one of those who thought it would be positively deplorable to move St. Bartholomew's Hospital from the City of London. It would give him much satisfaction to identify himself with their appeal, to which, no doubt, the public would readily respond. He would willingly grant the use of the Mansion House for the proposed meeting and to help them in any way they thought desirable.

It was arranged that the meeting should be held at the Mansion House on Tuesday, January 26, at 3 o'clock.

Sir Trevor Lawrence thanked the Lord Mayor on the part of the deputation, which then withdrew.

